# Predicted mechanisms of resistance to mTOR inhibitors

**DOI:** 10.1038/sj.bjc.6603353

**Published:** 2006-09-05

**Authors:** R T Kurmasheva, S Huang, P J Houghton

**Affiliations:** 1 Department of Molecular Pharmacology, St. Jude Children's Research Hospital, 332 N. Lauderdale, Memphis, TN 38105-2794, USA; 2 Department of Biochemistry & Molecular Biology, Louisiana State University Health Sciences Center, 1501 Kings Highway, Shreveport, LA 71130-3932, USA

**Keywords:** rapamycin, kinase inhibitors, mammalian target of rapamycin (mTOR), resistance mechanisms, cap-dependent translation, clinical resistance

## Abstract

The serine/threonine kinase, mTOR (mammalian Target of Rapamycin) has become a focus for cancer drug development. Rapamycins are highly specific inhibitors of mTOR and potently suppress tumour cell growth by retarding cells in G1 phase or potentially inducing apoptosis. Currently, both rapamycin and several analogues are being evaluated as anticancer agents in clinical trials. Results indicate that many human cancers have intrinsic resistance and tumours initially sensitive to rapamycins become refractory, demonstrating acquired resistance. Here, we consider mechanisms of resistance to inhibitors of mTOR.

The mammalian target of rapamycin, mTOR is a serine/threonine kinase evolutionarily conserved from yeast to human in the catalytic domain. As the C-terminus of mTOR is highly homologous to the catalytic domain of phosphatidylinositol 3 kinase (PI3K), mTOR is considered a member of PI3K-related kinase family (designated PIKK). The role of mTOR in cell growth, metabolism, and cancer has been reviewed extensively (Bjornsti and Houghton, 2004; Wullschleger *et al*, 2006). Signalling pathways upstream and downstream of mTOR are presented in Figure 1. Increasing evidence has implicated mTOR as a sensor that integrates extracellular and intracellular events, coordinating cell size (growth), proliferation, and survival. Mammalian target of rapamycin may directly or indirectly regulate translation initiation (through the mTOR-raptor [TORC1] complex), actin organisation (through the mTOR-rictor [TORC2] complex), membrane trafficking, protein degradation, ribosome biogenesis and tRNA synthesis, as well as transcription. Mammalian target of rapamycin signalling is negatively regulated by amino-acid deficiency and increased AMP levels, suppressing cap-dependent protein synthesis. Thus, mTOR in concert with tuberin and hamartin proteins that form the tuberous sclerosis complex (TSC) and Rheb, appears to sense nutrient and growth factor status, as well as energy charge, and regulates progression from G1 to S phase.

## CLINICAL DEVELOPMENT OF RAPAMYCIN ANALOGUES

**Figure 1:**
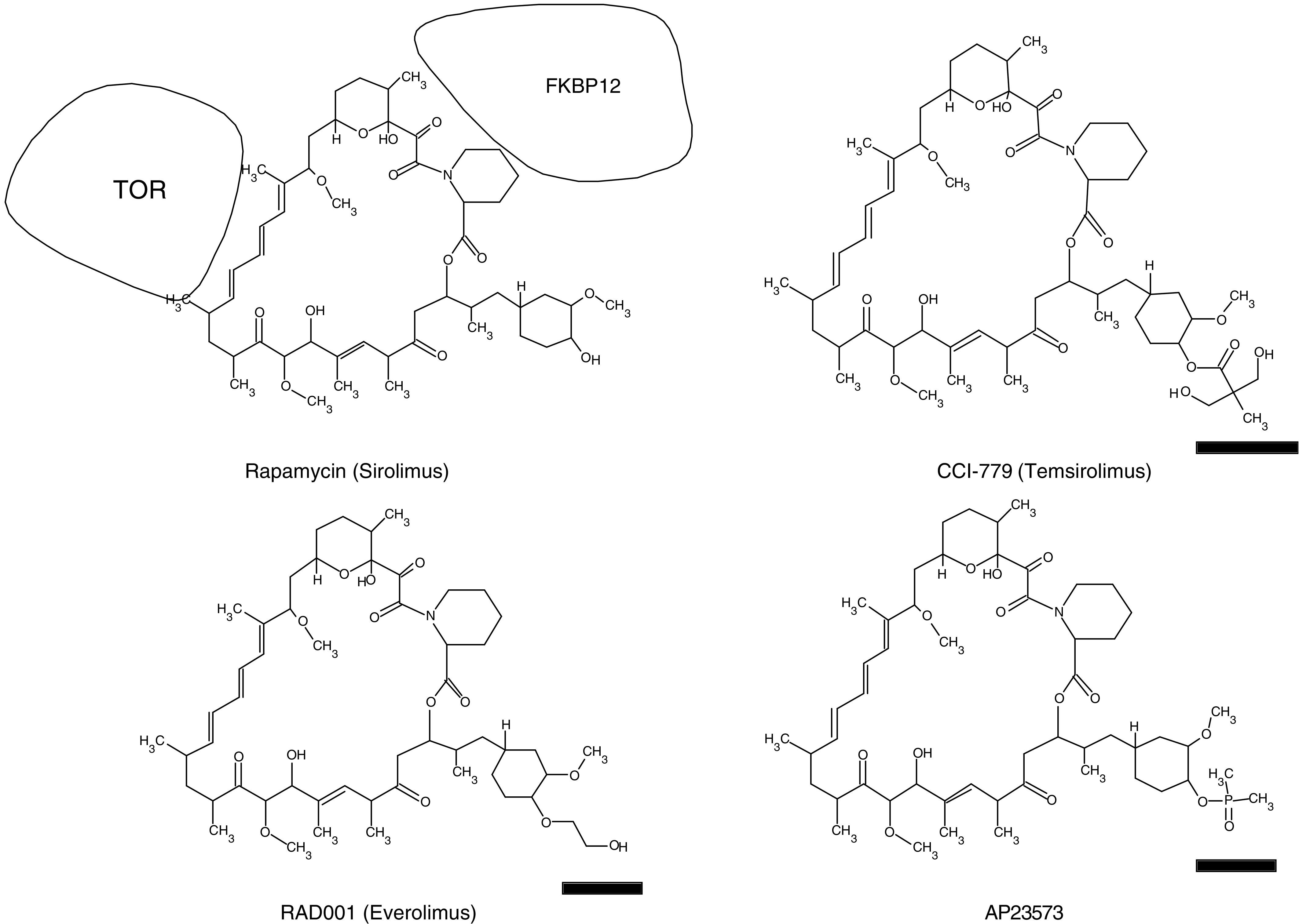
Chemical structure of rapamycin and its analogues currently in clinical trials as anticancer chemotherapeutic agents. Bars indicate the chemical modifications to rapamycin.

A number of clinical trials with the rapalogues (CCI-779, RAD001, and AP23573; Figure 1) have now been completed. The phase I trial evaluating the safety of CCI-779 was examined for both daily and weekly i.v. treatment in patients with a number of different types of tumours (Dancey, 2002). Objective responses (non-small-cell carcinoma, neuroendocrine, and breast carcinomas), and minor responses or stable disease (cervical carcinoma, uterine carcinoma, renal cell carcinoma, and soft tissue sarcoma) were observed. Phase II trials for renal cell carcinoma yielded an objective response rate of 5–7%, a minor response rate of 26–29%, and stable disease in approximately 40% of the patients (Atkins *et al*, 2004). Treatment is associated with an increase in survival time of approximately 4 months. For Mantle cell lymphoma, an overall response rate of 38% (3% complete response and 35% partial response), with a median time to progression of 6.9 months in responders *vs* 6.5 months for all patients treated (Witzig *et al*, 2005). CCI-779 appears to have significant activity in endometrial cancer, irrespective of PTEN (phosphatase and tensin homologue deleted on chromosome ten) status, yielding objective responses in 25% of patients, and causing disease stabilisation in over half of the patients on study. Median progression-free survival was 8 months. For AP23573, phase II results suggest significant activity in approximately 30% of patients with previously treated subtypes of sarcoma. Thus, in these studies there is clear evidence of acquired resistance where tumour growth is initially arrested but where relapse occurs on treatment with rapamycin analogues. In contrast, the majority of patients with advanced breast cancer (overall response rate of 9.2%) appear to exhibit intrinsic resistance to this class of agent (Chang *et al*, 2005).

## MECHANISM OF ACTION OF RAPAMYCINS

Rapamycin and its analogues in clinical development are highly specific inhibitors of mTOR, and differ only slightly in chemical structure (Figure 1) that results in improved chemical stability and pharmaceutical properties. Rapamycins bind a *M*_r_ 12 000 cytosolic immunophilin termed FK-binding protein (FKBP-12). The FKBP–rapamycin complex binds to the FK–rapamycin binding (FRB) domain of mTOR, resulting in inhibition of the function of mTOR in the TORC1 complex. Whether rapamycin or FKBP–rapamycin complex directly inhibits the kinase activity of mTOR is controversial, rather FKBP–rapamycin complex may disrupt higher order mTOR–protein complexes, perhaps displacing substrates from the catalytic domain (Bjornsti and Houghton, 2004). As each of the rapalogues in clinical development has an essentially identical mechanism of action, it is probable that resistance mechanisms will be similar.

## RAPAMYCIN-SENSITIVE SIGNALLING PATHWAYS MEDIATED BY MTOR

4E-BP1 and S6K1 are the best characterised downstream effector molecules of mTOR. 4E-BP1 functions as a suppressor of eukaryotic initiation factor 4E (eIF4E), the mRNA cap-binding protein. In response to mitogens, six sites of 4E-BP1 can be phosphorylated (Shah *et al*, 2000). In the presence of rapamycin, 4E-BP1 becomes hypo-phosphorylated and associates with eIF4E, preventing binding of eIF4E to the scaffold protein eIF4G and formation of the eIF4F pre-initiation complex required for cap-dependent translation of mRNA. As a result, rapamycin may downregulate mTOR-controlled synthesis of essential proteins involved in cell cycle progression, such as cyclin D1 and ornithinine decarboxylase, and survival (hypoxia inducible factor 1*α*, and c-MYC).

S6K1 and S6K2, downstream of mTOR, are both inhibited by rapamycin (Jefferies *et al*, 1997). Similar to 4E-BP1, activation of S6K1 requires hierarchical phosphorylation. *In vitro*, mTOR phosphorylates only Thr389 in the regulatory domain. A recent model proposed by the Blenis laboratory suggests that S6K1 is phosphorylated in the pre-initiation complex by mTOR leading to dissociation and full activation of S6K1 by PDK1. Activated S6K1 phosphorylates eIF4B, which then binds in the pre-initiation complex (Holz *et al*, 2005).

## REGULATION OF SENSITIVITY TO MTOR INHIBITION BY SIGNALLING PATHWAYS

Phosphatase and tensin homologue deleted on chromosome ten (PTEN) is a dual-specificity protein phosphatase, and functions as a major negative regulator of the PI3-kinase/Akt signalling pathway. Loss of PTEN occurs frequently in human tumours (Vivanco and Sawyers, 2002), resulting in hyperphosphorylation or activation of Akt. Activated Akt positively regulates mTOR-dependent pathways through inhibition of the TSC complex, and forms the basis for rapamycin hypersensitivity of PTEN-deficient or -mutant cells. Either loss of PTEN or activation of Akt resulted in an increased sensitivity to the rapalogue CCI-779 in a panel of brain, prostate, breast cancer cell lines, and human tumours implanted in athymic nude mice (Yu *et al*, 2001). Most CCI-779-resistant tumour cells had a low or moderate level of activated Akt, although there are exceptions where loss of PTEN is not associated with hypersensitivity to CCI-779. In contrast to CCI-779 resistant lines, sensitive breast cancer cell lines were estrogen-dependent or lacked expression of the tumour suppressor PTEN, and/or overexpressed the Her-2 oncogene. One proposed mechanism by which low Akt activity induces rapamycin resistance is through allowing continued cap-independent protein synthesis of cyclin D1 and c-MYC proteins. The cyclin D1 mRNA 5′ untranslated region contains an internal ribosome entry site (IRES) and both this IRES and the c-*MYC* IRES are negatively regulated by Akt activity, and the function of both IRES's is enhanced following exposure to rapamycin. Hence, continued IRES-mediated translation initiation may permit cell cycle progression upon mTOR inactivation in cells in which Akt kinase activity is relatively low (Shi *et al*, 2005). Thus, one biomarker for intrinsic rapamycin resistance may be low levels of activation of Akt. On the other hand, rapamycin sensitivity of breast cancer lines correlated inversely with phospholipase D (PLD) activity. Elevating PLD activity led to rapamycin resistance, and inhibition of PLD activity increased rapamycin sensitivity. This is consistent with the notion that PLD-generated phosphatidic acid competes for rapamycin–FKBP binding to mTOR. In yeast, activation of the RAS/cAMP signalling pathway also confers pronounced resistance to rapamycin (Schmelzle *et al*, 2004); however, this association has not been reported for mammalian cells.

## MUTATIONS IN FKBP12 AND MTOR

Although direct inhibitors of mTOR kinase activity are being developed, most data on mechanisms of resistance apply to rapamycins. Rapamycin first binds FKBP-12 in mammalian cells. Either specific mutations in FKBP12 that prevent the formation of FKBP–rapamycin complex, or mutations in the FRB domain of mTOR that decrease the affinity of binding of the FKBP–rapamycin complex would cause rapamycin resistance. Such mutations were first found in the yeast *Saccharomyces cerevisiae*, where deletion of the RBP1 gene (a homologue of mammalian FKBP-12) resulted in recessive resistance to rapamycin, and expression of RBP1 restored rapamycin sensitivity. In mouse mast cells, mutations in FKBP-12 that substantially reduced binding affinity of FKBP-12 for rapamycin, rendered rapamycin resistance (Fruman *et al*, 1995). Resistance to rapamycin selected after mutagenesis is related to a dominant phenotype consistent with mutation in the FRB domain of mTOR (Dumont *et al*, 1995). Expression of an FRB-mutant mTOR (Ser2035 → Ile), having reduced affinity for binding the FKBP–rapamycin complex, confers high-level resistance (Dumont *et al*, 1995), Figure 2A.

## DYSREGULATION OF EIF4E

4E-BP1, the suppressor of eIF4E, has been widely recognised as the other primary downstream effector of mTOR. Acquired resistance to rapamycin was associated with decreased levels (∼10-fold) of 4E-BP1, the suppressor of eIF4E (Dilling *et al*, 2002) owing to decreased translation of 4E-BP1. In cells that reverted to rapamycin sensitivity, total levels of 4E-BP1 became similar to those in parental cells, and 4E-BP1 bound to eIF4E had similar response to serum starvation and IGF-I stimulation as found in parental cells. These data suggest that decrease of 4E-BP1 expression results in dysregulation of eIF4E, conferring rapamycin resistance.

**Figure 2:**
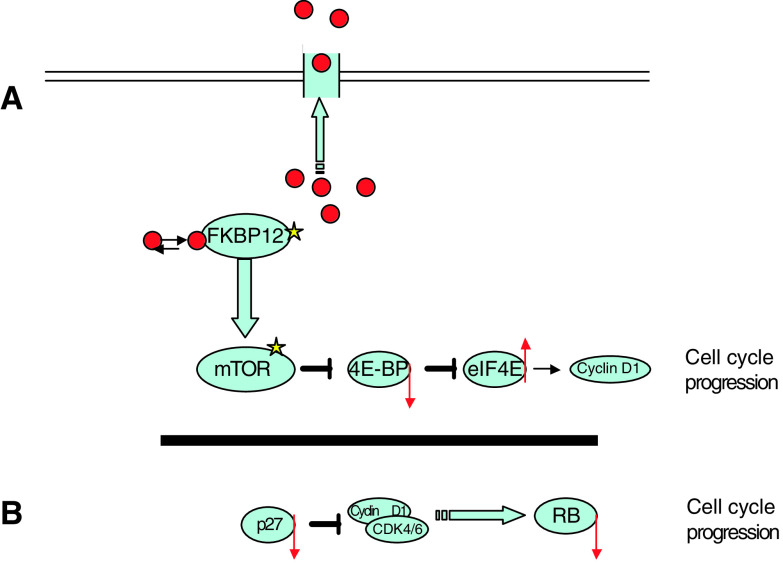
(**A**) Predicted mechanisms of resistance to rapamycin analogues. Rapamaycin or its derivatives (red balls) can be eliminated from cells by ABC transporters such as P-glycoprotein. Mutations of FKBP or mTOR (yellow stars) confer resistance. Acquired resistance to rapamycin has been associated with decreased stoichiometry between 4E-BP and eIF4E, either through decreased translation of 4E-BP or overexpression of eIF4E. (**B**) Inhibition of mTOR leads to decreased translation of cyclin D1 mRNA, and reduced levels of cyclin D1. In many cells, there is a concomitant stabilisation of the cyclin-dependent kinase inhibitor p27^Kip1^, and inhibition of CDK-cyclin activity, and decreased phosphorylation of RB. Cells deficient in p27^Kip1^ are partially resistant, whereas RB-null cells are completely resistant to inhibition of proliferation by rapamycin.

These results indicate that the rapamycin-regulated eIF4E pathway is crucial in inducing growth arrest, and dysregulation of eIF4E may facilitate the malignant phenotype. This is supported by clinical observations that dysregulation of the eIF4E pathway is associated with tumour progression (Nathan *et al*, 1997). In addition to decreased 4E-BP1 expression, increased eIF4E expression in mice is tumorigenic and increases the rate of tumour progression. When 4E-BP1 is overexpressed in highly resistant HCT8 cells (IC_50_>10 000 ng ml^−1^), they become sensitive (IC_50_ <10 ng ml^−1^) to rapamycin (Dilling *et al*, 2002). Potentially, the ratio of 4E-BP to eIF4E may determine whether inhibition of mTOR elicits a biologically significant tumour response (Figure 2A).

## DEFECTIVE REGULATION OF THE RETINOBLASTOMA (RB) PROTEIN CHECKPOINT

In malignant cells, the RB checkpoint can be bypassed by loss of RB protein or function, loss of p27^Kip1^, and overexpression of cyclin E or other cyclins. The role of p27^Kip1^ in modulating rapamycin sensitivity (Luo *et al*, 1996) implies that mTOR has a role in controlling the phosphorylation state of RB, however, this may be cell-context specific. Rapamycin prevents mitogen-stimulated downregulation of the cdk inhibitor p27^Kip1^ and suggests that p27^Kip1^ is involved in the antiproliferative activity of rapamycin. Prolonged culture of sensitive cells in the presence of drug resulted in rapamycin-resistant clones that exhibited low p27^Kip1^ protein, no decrease in mRNA level but a high rate of ubiquitin-independent degradation. p27^Kip1^ in resistant cells could not be regulated either in response to serum or rapamycin, and as a result, RB phosphorylation was not blocked. However, only partial resistance to antiproliferative activity of rapamycin was found in p27^−/−^ mouse embryo fibroblasts (MEFs) and p27^−/−^ splenic T lymphocytes, indicating that there exist p27-dependent and -independent pathways that determine rapamycin sensitivity (Figure 1B). Rapamycin also inhibits insulin-induced RB phosphorylation in 3T3-L1 adipocytes but not in rapamycin-sensitive NIH3T3 fibroblasts where rapamycin does not alter the phosphorylation status of RB or two RB-related proteins p107 and p130. Similarly, in Ba/F3 p210 cells expressing BCR/ABL, rapamycin arrested growth, but failed to alter the hyperphosphorylation of RB protein (Gesbert *et al*, 2000). RB-null MEFs, however, are highly resistant to rapamycin (IC_50_ >10 *μ*g ml^−1^) relative to wild-type cells (IC_50_∼1 ng ml^−1^) (unpublished data) (Figure 2B).

## DEFECTIVE REGULATION OF THE P53 CHECKPOINT

p53 is a tumour suppressor and transcription factor that has been found to be mutated in ∼50% of human cancers and p53 status may also determine the cellular response to rapamycin (Huang *et al*, 2003). When cultured under autocrine conditions (serum-free) p53^+/+^ MEFs exposed to rapamycin accumulate in G1 phase and maintain viability, whereas p53-mutant cells accumulate in G1 phase and undergo apoptosis. In p53-defective cells, inhibition of mTOR leads to a rapid and maintained induction of a stress response characterised by increased phospho-c-Jun, whereas in cells with functional p53 this stress response is suppressed (Huang *et al*, 2003). Mechanistically, inhibition of mTOR leads to decreased activity of protein phosphatase 5 (PP5) and activation of apoptosis signal regulating kinase 1. Rapamycin-induced apoptosis can be inhibited by expression of functional p53, by overexpression of PP5, decreased expression of 4E-BP1, or overexpression of Bcl2.

## MUTATIONS OF PP2A-RELATED PROTEIN PHOSPHATASES

Several serine/threonine protein phosphatases have been identified as the components of mTOR signalling pathway in mammalian cells (Dennis *et al*, 1999). Rapamycin resistance caused by mutations of PP2A-related phosphatases was first found in yeast. Studies in mammalian cells also indicate that association of *α*4 (the homologue of yeastTap42) with PP2A, PP4, and PP6 is related to rapamycin sensitivity (Murata *et al*, 1997). For example, in rapamycin-sensitive Jurkat cells, rapamycin dissociated *α*4 from PP2Ac, and transfection of mouse *α*4 into Jurkat cells conferred rapamycin resistance. In rapamycin-resistant Raji cells, rapamycin did not affect association of *α*4 with PP2Ac. However, other studies have not demonstrated rapamycin-induced dissociation of *α*4 from PP2A or PP6. Thus, at this time the significance of *α*4 to rapamycin resistance is controversial.

## MUTATIONS OF ATM

ATM (*a*taxia *t*elangiectasia, *m*utated) cells have been reported to be rapamycin resistant. In contrast, early passage murine embryo fibroblasts derived from ATM^−/−^ mice are not resistant to rapamycin (Germain and Houghton, unpublished data) distinguishing the null phenotype from the ATM mutant cells. As ATM cells have genetic instability, it is possible that resistance to rapamycin is a consequence of additional mutations and not related directly to defects in ATM signalling.

## MUTATIONS OF 14-3-3

In yeast, the two homologues of the mammalian 14-3-3 proteins act as multicopy suppressors of the growth-inhibitory phenotype caused by rapamycin (Bertram *et al*, 1998). Overexpression of either BMH1 or BMH2, or three human 14-3-3 isoforms (*β*, *τ*, and *η*) in yeast conferred rapamycin resistance, whereas disruption sensitised to rapamycin. The mechanism by which 14-3-3 proteins cause rapamycin resistance is not known; however, potentially 14-3-3 proteins may play a role in determining rapamycin sensitivity in mammalian cells.

## PREVENTION OF APOPTOSIS BY GROWTH FACTORS AND CYTOKINES

Substantial data support a role for mTOR signalling through the eIF4E pathway in promoting cell survival, although exogenous factors may modulate the apoptotic response to mTOR inhibition. IGF-I or insulin protect from rapamycin-induced apoptosis (Kurmasheva and Houghton, 2006) by activating Akt-dependent and -independent pathways resulting in phosphorylation and inactivation of the proapoptotic protein Bad, thus promoting the activity of Bcl2. Overexpression of Bcl2 also confers resistance to rapamycin-induced cell killing.

Anecdotally, in many cell lines and patient tumour biopsies (O’Reilly *et al*, 2006), inhibition of mTOR activates Akt and inhibits downstream substrates (FoxO transcription factors, Bad, and GSK3*α*/*β*). Increased phospho-Akt was correlated with increased levels of IRS-1 but not IRS-2. IRS-1 is phosphorylated by S6K1 at Ser312 resulting in its dissociation from the IGF-1 receptor, and proteasome-mediated degradation (Shi *et al*, 2005). Inhibition of mTOR inactivates S6K1 and stabilises IRS-1 associated with receptors, thus alleviating this negative regulation. Inhibition of IGF-1R abrogated rapamycin-induced phospho-Akt induction and substrate phosphorylation, and combination of rapamycin with an inhibitor of IGF-1R had additive effects on proliferation irrespective of PTEN status and increased apoptosis in a PTEN mutant cancer cell line (O’Reilly *et al*, 2006). Thus, either exogenous IGF-1 or derepression of IRS-1 in the presence of rapamycin may induce resistance to growth inhibition or apoptosis induced by mTOR inhibition (see Easton *et al*, 2006). Similarly IL-3 induces expression of Pim-2 and confers resistance to rapamycin, probably through the dual action of phosphorylating 4E-BP1 and Bad. Pim kinases are components of the transcriptional response to cytokines or antigen receptor ligation, and overexpression of Pim-1 or Pim-2 can maintain 4E-BP1 phosphorylation (Ser65) in the presence of rapamycin, conferring resistance. Consistent with the role of Pim kinases modulating the activity of mTOR inhibitors, Pim-2^−/−^ or Pim-1^−/−^Pim-2^−/−^ cells are hypersensitive to rapamycin (Fox *et al*, 2005).

## INHIBITION OF MTOR IN TORC2 COMPLEXES

Mammalian target of rapamycin complexed with rictor (TORC2) is thought to be insensitive to rapamycin, and to play a role in regulating the actin cytoskeleton (Wullschleger *et al*, 2006). The TORC2 complex phosphorylates Akt at Ser473, and consequently may regulate both survival through Akt, and also TORC1 (mTOR-raptor) activity. Prolonged inactivation of TORC1 by rapamycin potentially leads to a shift of mTOR from the TORC2 complex to being rapamycin-bound, and lead to decreased Akt activation, activation of GSK-3*β* (which regulates cyclin D1 proteolysis, and enhances rapamycin-mediated growth inhibition), and apoptosis. Thus, for direct inhibitors of mTOR kinase, the potential for upregulation of Akt via the IRS-1 pathway and protection from cell death seems less likely. The TORC2 complex will also be targeted by direct mTOR kinase inhibitors that bind into the ATP pocket of the catalytic domain. By analogy to acquired resistance mechanisms to imatinib or gefitinib that frequently are caused by mutations in ATP-binding site for BCR-ABL and ERBB1, respectively, it is probable that ATP-mimetic mTOR inhibitors will select for binding site mutants.

## ABC-TRANSPORTERS

The ability of cyclosporin A, FK506, and rapamycin to overcome P-glycoprotein-mediated multidrug resistance has been demonstrated (Arceci *et al*, 1992). The multidrug resistance-reversing activity increased in the order rapamycin <FK506 < cyclosporin A, irrespective of whether the resistant cells overexpressed hamster or human P-glycoprotein. The interaction of the three macrolides with P-glycoprotein was characterised by their ability bind with high affinity to P-glycoprotein, and to competitively inhibit the photoaffinity labelling of plasma membranes of drug-resistant cells by iodomycin. Although an inhibitor of P-glycoprotein activity rapamycin decreased the rate of ATP hydrolysis with respect to the basal rate, it did not completely inhibit the activity. Hence, rapamycin can be classified as a substrate for transport by P-glycoprotein (Kerr *et al*, 2001), as a consequence tumours overexpressing P-glycoprotein (MDR1) may be resistant to rapamycins (Figure 1A).

## ANGIOGENESIS-ASSOCIATED RESISTANCE MECHANISMS

Guba *et al* (2002) first reported the anecdotal result where cells were resistant to rapamycin *in vitro*, but tumours from the same cells in mice were growth inhibited by rapamycin treatment. They concluded that the antitumor activity of rapamycin was due to its antiangiogenic activity as rapamycin decreased production of vascular endothelial growth factor (VEGF) and markedly inhibited response of vascular endothelial cells to stimulation by VEGF. Similar results have been obtained with human cancer cell lines that are intrinsically resistant to rapamycin analogues *in vitro*, but where *in vivo* growth of tumour xenografts is inhibited significantly. The mTOR pathway controls cap-dependent translation, and hence potentially levels of hypoxia inducible factor 1*α* (HIF-1*α*), cellular proliferation, and migration. Thus, inhibition of mTOR may have direct effects on cancer cell proliferation and survival, indirect effects *via* inhibition of HIF-1*α*, thus reducing tumour-elicited VEGF, direct effects on vascular endothelial cells, or vascular smooth muscle cells (Humar *et al*, 2002; Majumder *et al*, 2004). For example, induction of HIF-1*α* and VEGF by the CML-associated oncogene, BCR-ABL, is mTOR-dependent (Mayerhofer *et al*, 2002), and *in vitro*, rapamycin inhibited VEGF production in primary cultures from BCR-ABL transformed, imatinib resistant, CML (Mayerhofer *et al*, 2005). The role for mTOR in VEGF production is supported by regulation of HIF-1*α* by mTOR signalling and increased VEGF in cells deficient in the TSC that negatively regulates mTOR via Rheb (Hudson *et al*, 2002). However, other studies support a role mainly for PI3K and to a lesser extent mTOR being required for insulin-induced HIF-1*α* expression (Treins *et al*, 2002). Our studies indicate that rapamycin treatment has little effect on hypoxia-driven VEGF production in most rhabdomyosarcoma or neuroblastoma cell lines (Kurmasheva *et al*, submitted). Thus, in these cells it is unlikely that rapamycin would block tumour-derived VEGF, although it may directly block the response of vascular endothelial or other stromal cells in tumour tissue. Potentially, *in vivo* resistance to mTOR inhibition could be elicited by secretion of angiogenic factors that signal to stromal cells *via* mTOR-independent pathways to increase proliferation or motility of vascular cells.

## CONCLUSIONS

The mechanisms for resistance to rapamycin analogues are likely to be complex. In part, it remains unclear exactly why inhibition of mTOR signalling results in growth inhibition, as inhibition of mTOR signalling appears similar in rapamycin-sensitive or -resistant cell lines. It is also probable that resistance mechanisms will be similar for the rapalogues currently in clinical development. The mechanism(s) for resistance to catalytic kinase inhibitors, however may more likely relate to mTOR mutations within the ATP binding site as identified for imatinib and gefitinib. Although preclinical models can suggest potential mechanisms for resistance to rapamycins, it is of critical importance to characterise tumour biopsy tissue at initiation of treatment and at subsequent relapse in order to ascertain which mechanisms apply to clinical cancer.
